# Hyponatremia After Colonoscopy Bowel Preparation: Challenges in Managing Syndrome of Inappropriate Antidiuretic Hormone Without Established Guidelines

**DOI:** 10.7759/cureus.61960

**Published:** 2024-06-08

**Authors:** Talal Alomar, Sharon Choe, Deepti Boddupalli

**Affiliations:** 1 Internal Medicine, Creighton University School of Medicine, Phoenix, USA

**Keywords:** treatment of hyponatremia, polyethylene glycol, colonoscopy complications, bowel prep, syndrome of inappropriate secretion of antidiuretic hormone (siadh)

## Abstract

Syndrome of inappropriate antidiuretic hormone secretion (SIADH) is a condition that leads to free water retention and solute excretion, predisposing patients to hyponatremia. We present the case of a 79-year-old female with a history of SIADH well-controlled with fluid restriction and sodium chloride tablets who presented with hyponatremia after bowel preparation. Her medication regimen was not adjusted before she took the bowel preparation. Her SIADH diagnosis was unknown when she presented but was exemplified by her sodium levels dropping while on a normal saline drip on her third day in the hospital. She was able to successfully take the bowel preparation without hyponatremia after oral urea was added to her regimen.

There are currently no clinical guidelines for SIADH patients receiving bowel preparation for colonoscopies and no case reports describing this situation. We discuss the pathophysiology behind the patient’s fluctuating sodium levels when on various maintenance fluids and when on fluid restriction. This case concludes that it is imperative to either increase solute intake or increase free water excretion for SIADH patients receiving bowel preparation to prevent potentially deadly hyponatremia.

## Introduction

There are two water retention systems in the body namely antidiuretic hormone (ADH) and aldosterone. ADH is synthesized by the hypothalamus and secreted by the posterior pituitary, also known as the neurohypophysis. ADH upregulates the number of aquaporins in tubular collecting duct cells to increase free water reabsorption [1]. Aldosterone is produced in the adrenal cortex and increases sodium and water retention. Syndrome of inappropriate antidiuretic hormone secretion (SIADH) occurs when ADH is constantly secreted, causing an inappropriate increase in free water retention. SIADH is associated with small cell lung cancer and other malignancies, pneumonia, and various chemotherapy medications [1]. The lack of water excretion causes concentrated urine with hypotonic/diluted solutes in the blood. SIADH patients are normally euvolemic because aldosterone is downregulated, leading to sodium and water excretion. The net effect of high ADH and low aldosterone is sodium loss, which causes hyponatremia. SIADH patients are treated with free water restriction, sodium chloride or salt tablets, and medications that increase free water loss such as oral urea, demeclocycline, and conivaptan/tolvaptan.

Understanding the intricate interplay between water retention systems in the body sheds light on potential complications arising from medical procedures. The implications of SIADH extend beyond endocrine disorders. Notably, the risk of electrolyte disturbances, including hyponatremia, becomes particularly pertinent in medical interventions such as bowel preparation for diagnostic procedures like colonoscopy.

Thorough bowel cleansing is a prerequisite for colonoscopy, which is the gold standard for diagnostic screening of colonic diseases. The most commonly used intestinal cleansing agents utilize polyethylene glycol (PEG), which is considered to be the safest among other options [2]. However, PEG can still cause adverse effects, especially in individuals with risk factors like older age, chronic kidney disease, heart failure, history of electrolyte disturbances, or use of thiazide diuretics and selective serotonin reuptake inhibitors [3]. Hyponatremia has been described as a risk of bowel preparation and can lead to seizure, encephalopathy, and coma when severe [4]. A literature review of 16 cases of symptomatic hyponatremia after bowel preparation revealed that 11 patients presented with seizures [5]. Patients with known hyponatremia, such as patients with SIADH, may require medication adjustments prior to undergoing bowel preparation to prevent complications. Herein, we present a patient with a prior history of SIADH who developed symptomatic hyponatremia after bowel preparation.

## Case presentation

A 79-year-old female presented to the emergency department (ED) with one day of dizziness, nausea, leg weakness, and muscle cramps. One day prior to the presentation, the patient had consumed GoLytely bowel preparation and sports drinks in preparation for a planned upper endoscopy and colonoscopy to evaluate for chronic dysphagia. She reported numerous episodes of watery diarrhea due to the bowel preparation. Due to chronic postprandial epigastric pain and dysphagia, the patient reported she only ate soft foods and soups in very small portions.

Her medical history was significant for type 2 diabetes mellitus treated with metformin, hypertension, and a one-year history of chronic hyponatremia. She was compliant with sodium chloride tablets for her hyponatremia. The patient reported her baseline sodium levels were approximately 130 mmol/L. On examination, the patient was afebrile and slightly hypertensive. She had dry mucous membranes, indicating a hypovolemia superimposed on baseline SIADH post-bowel preparation.

Investigations

Initial laboratory investigations (Table 1) revealed significant hyponatremia (118 mmol/L, reference range: 135-145) with a low serum osmolality of 254 mOsm/kg (reference range: 275-300), indicating hypotonic hyponatremia. In addition, testing revealed the patient was in ketoacidosis with a beta-hydroxybutyrate level of 21.1 mg/dL (reference range: 0-2.18), likely secondary to chronic starvation. Laboratory investigations revealed potassium 3.7 mmol/L (reference range: 3.6-5.3), chloride 84 mmol/L (reference range: 100-110), bicarbonate 18 mmol/L (reference range: 19-27), anion gap 16 mmol/L (reference range: 7-15), glucose 168 mg/dL (reference range: 65-99), blood urea nitrogen 5 mg/dL (reference range: 8-25), calcium 8.9 mg/dL (reference range: 8.5-10.3), urine sodium 71 mmol/L, urine osmolality 299 mOsm/kg, and urine creatinine 16.78 mg/dL. The electrocardiogram was unremarkable. The elevated urine osmolality in the setting of hypovolemia favored a diagnosis of SIADH.

**Table 1:** Serum and urine chemistries Note: Abnormal values are shown in bold.

Serum chemistry	Lab value	Reference range
Sodium	118 mmol/L	135-145 mmol/L
Potassium	3.7 mmol/L	3.6-5.3 mmol/L
Chloride	84 mmol/L	100-110 mmol/L
Calcium	8.9 mg/dL	8.5-10.3 mg/dL
Blood urea nitrogen (BUN)	5 mg/dL	8-25 mg/dL
Bicarbonate	18 mmol/L	19-27 mmol/L
Anion gap	16 mmol/L	7-15 mmol/L
Osmolality	254 mOsm/kg	275-300 mOsm/kg
Glucose	168 mg/dL	65-99 mg/dL
Beta hydroxybutyrate	21.1 mg/dL	0-2.18 mg/dL
Urine chemistry		
Urine sodium	71 mmol/L	<40 mmol/L
Urine osmolality	299 mOsm/kg	<100 mOsm/kg
Urine creatinine	16.78 mg/dL	No reference value

Treatment

**Figure 1:**
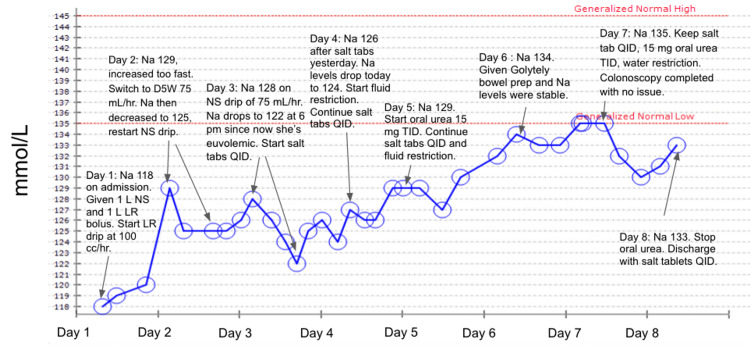
Graph of the patient’s sodium levels over eight days with labels indicating interventions for each day Na: sodium; NS: normal saline; LR: lactated Ringer’s; D5W: dextrose 5% in water

In the ED, the patient received 1 L of normal saline (NS) and 1 L of lactated Ringer’s (LR) followed by maintenance intravenous (IV) fluids of LR drip at 100 ml/hour. Her sodium increased to 129 mmol/L the next day, so her fluids were switched to 75 ml/hour of 5% dextrose in water (D5W) to slow correction, and an NS drip of 75 ml/hour was started a few hours later. The next day her sodium dropped to 122 mmol/L, despite being on the NS drip. She was started on sodium chloride tablets three times daily and had a sodium of 124-126 mmol/L the next day. Due to her consistently low sodium levels, a more appropriate SIADH treatment regimen of sodium chloride tabs four times daily with a fluid restriction of 1.2 liters daily was started. The following day, her sodium increased to 129 mmol/L, which was still below reference ranges. She was started on oral urea 15 mg three times daily on day five of admission with continuation of sodium chloride tablets and water restriction. By day six of admission, her sodium had reached 134 mmol/L, and she was restarted on bowel preparation without subsequent disturbance of her sodium. Her hyponatremia normalized by day seven of admission (Figure 1), and she underwent endoscopy and colonoscopy without complications. The endoscopy revealed an upper esophageal benign stricture, likely the cause of her dysphagia. The colonoscopy showed severe sigmoid diverticulosis with large internal hemorrhoids. Gastroenterology recommended a high-fiber diet and no further colon cancer screening. Her ketoacidosis improved during admission with fluids, and her nausea was controlled with ondansetron. She was discharged home on sodium chloride tablets four times daily with a stable sodium level.

## Discussion

Affecting approximately 5% of adults and 35% of hospitalized patients, hyponatremia is the most common electrolyte disturbance [6]. Mild symptoms and signs of hyponatremia include weakness and nausea while severe hyponatremia, a medical emergency, can present with seizures, coma, or death [6]. SIADH, a euvolemic hyponatremia characterized by an excess release of ADH, accounts for one-third of all cases of hyponatremia. SIADH was first described by Schwartz et al. in 1957 in a study analyzing severe hyponatremia in the setting of excellent renal function in two patients with lung tumors [7]. Though SIADH can be caused by genetic mutations in renal V2 receptors, it most commonly arises as a secondary condition of another disease process including central nervous system disturbances, malignancies, drugs, surgeries, hormone deficiencies, and hormone administration [8]. SIADH is diagnosed through laboratory studies that show hypotonic hyponatremia combined with urine studies showing elevated urine sodium levels and inappropriately elevated urine osmolality (mOsm/kg) [9].

There are no guidelines on how to prepare patients with chronic hyponatremia for bowel cleansing routines. In our case, there was no adjustment to the patient’s regime which led to her complications. Symptomatic hyponatremia is a known risk of bowel preparation for colonoscopy [4], so extra caution is warranted in patients with chronic hyponatremia like our patient. GoLYTELY, in addition to its main ingredient of PEG, contains sodium in the form of sodium sulfate, sodium bicarbonate, and sodium chloride. Yet, there still remains a risk for electrolyte disturbance. The combination of diffuse diarrhea and the lack of food during bowel preparation facilitates a rapid loss of sodium, illustrated by our patient who became hyponatremic even while taking sodium chloride tablets and consuming sports drinks. Some type of medication adjustment to increase free water excretion (oral urea, demeclocycline, conivaptan/tolvaptan) or to increase the number of scheduled salt tablets may prevent such complications.

When correcting hyponatremia in a patient with SIADH, it is important to assess volume status. Our patient’s sodium level reached a plateau on day three of admission due to her transition from a hypovolemic to a euvolemic state. In a state of hypovolemia, aldosterone is activated by the renin-angiotensin-aldosterone system (RAAS) which increases reabsorption of sodium. In a euvolemic state, the RAAS is no longer activated while ADH is continually activated, and all of the solutes from IV fluids are essentially excreted. More water is absorbed into the body than excreted out, exacerbating existing hyponatremia. Since starting the patient on salt tablets four times a day did not produce satisfactory results, we started additionally started her on urea, an osmotic diuretic, to increase free water excretion. Current data on the use of urea for SIADH is limited, and a recent systematic review found no definite evidence for the use of urea as a treatment for SIADH [10]. However, our patient showed significant improvement with urea, providing anecdotal support for the consideration of oral urea in refractory SIADH.

To avoid detrimental complications, we suggest increasing the oral sodium chloride dosage or adding urea in patients with chronic hyponatremia prior to bowel preparation. Split-dose regimens, consisting of consuming half of the laxative the night before the colonoscopy and the other half on the day of the procedure, may also be considered as it has been reported to be superior to day-before regimens in quality of colon cleansing and patient preference [11].

## Conclusions

SIADH causes euvolemic hypotonic hyponatremia with elevated urine osmolality. Treatment includes increasing solute intake with salt tablets or increasing free water excretion with oral urea, demeclocycline, or conivaptan/tolvaptan.

This is the first known case report of a patient with SIADH who successfully underwent a colonoscopy. There are no guidelines on how to adjust medication regimens for SIADH patients who ingest bowel preparation prior to colonoscopy. Our case represents a successful treatment of a patient with SIADH by adding oral urea to her regimen before bowel preparation. This prevented her hyponatremia and allowed her to get the important colonoscopy procedure. We hope this case has increased awareness of this issue and can guide clinical decision-making for physicians in the future.
